# A Pilot Study on the Metabolic Impact of Mediterranean Diet in Type 2 Diabetes: Is Gut Microbiota the Key?

**DOI:** 10.3390/nu13041228

**Published:** 2021-04-08

**Authors:** Shámila Ismael, Marta P. Silvestre, Miguel Vasques, João R. Araújo, Juliana Morais, Maria Inês Duarte, Diogo Pestana, Ana Faria, José B. Pereira-Leal, Joana Vaz, Pedro Ribeiro, Diana Teixeira, Cláudia Marques, Conceição Calhau

**Affiliations:** 1 Nutrition and Metabolism, NOVA Medical School, Faculdade de Ciências, Universidade NOVA de Lisboa, 1169-056 Lisboa, Portugal; shamila.ismael@nms.unl.pt (S.I.); marta.silvestre@nms.unl.pt (M.P.S.); miguel.vasques@outlook.com (M.V.); joaoricardo.araujo@nms.unl.pt (J.R.A.); juliana.morais@nms.unl.pt (J.M.); maria.ines.duarte@edu.nms.unl.pt (M.I.D.); diogopestana@nms.unl.pt (D.P.); ana.faria@nms.unl.pt (A.F.); diana.teixeira@nms.unl.pt (D.T.); ccalhau@nms.unl.pt (C.C.); 2 CINTESIS—Center for Health Technology Services Research, NOVA Medical School, Faculdade de Ciências, Universidade NOVA de Lisboa, 1169-056 Lisboa, Portugal; 3 Unidade Universitária Lifestyle Medicine José de Mello Saúde by NOVA Medical School, 1169-056 Lisboa, Portugal; 4 Endocrinology Department, Centro Hospitalar e Universitário Lisboa Central, 1069-166 Lisboa, Portugal; 5 CHRC—Comprehensive Health Research Center, CEDOC—Chronic Diseases Research Center, NOVA Medical School, Faculdade de Ciências, Universidade NOVA de Lisboa, 1169-056 Lisboa, Portugal; 6 Ophiomics-Precision Medicine, 1600-513 Lisboa, Portugal; jleal@ophiomics.com (J.B.P.-L.); jvaz@ophiomics.com (J.V.); 7 Laboratory Medicine Center Germano de Sousa, 1600-513 Lisboa, Portugal; pedro.ribeiro@germanodesousa.com

**Keywords:** gut microbiota, Mediterranean diet, type 2 diabetes

## Abstract

The Mediterranean diet (MD) has been recommended for type 2 diabetes (T2D) treatment. The impact of diet in shaping the gut microbiota is well known, particularly for MD. However, the link between MD and diabetes outcome improvement is not completely clear. This study aims to evaluate the role of microbiota modulation by a nonpharmacological intervention in patients with T2D. In this 12-week single-arm pilot study, nine participants received individual nutritional counseling sessions promoting MD. Gut microbiota, biochemical parameters, body composition, and blood pressure were assessed at baseline, 4 weeks, and 12 weeks after the intervention. Adherence to MD [assessed by Mediterranean Diet Adherence Screener (MEDAS) score] increased after the intervention. Bacterial richness increased after 4 weeks of intervention and was negatively correlated with fasting glucose levels and Homeostatic Model Assessment for Insulin Resistance (HOMA-IR). *Prevotella* to *Bacteroides* ratio also increased after 4 weeks. In contrast, glycated haemoglobin (HbA1c) and HOMA-IR were only decreased at the end of study. Alkaline phosphatase activity was assessed in fecal samples and was negatively correlated with HbA1c and positively correlated with bacterial diversity. The results of this study reinforce that MD adherence results in a better glycemic control in subjects with T2D. Changes in gut bacterial richness caused by MD adherence may be relevant in mediating the metabolic impact of this dietary intervention.

## 1. Introduction

Strong evidence supports the efficacy and cost-effectiveness of individualizing nutrition therapy as a component of quality diabetes care for adults with diabetes [1]. Recently, dietary guidelines for the management of type 2 diabetes have started to focus on dietary patterns rather than isolated nutrients [2,3]. They recommend maintaining the pleasure of eating and providing the necessary tools to empower patients to establish healthy eating patterns themselves, rather than talking about unique foods or micro/macronutrients. Low-carbohydrate diet, Dietary Approaches to Stop Hypertension (DASH) diet, vegetarian diet, and Mediterranean diet are some of the dietary patterns recommended by American and European Guidelines for type 2 diabetes management. In this regard, the Mediterranean diet has been the most studied and is linked with greater glycemic control when compared to other patterns [2,4]. This dietary pattern is characterized by high consumption of plant-based foods, low-to-moderate consumption of meat products, use of herbs and spices as substitutes for salt, and use of olive oil as the main type of fat; it is also characterized by cooking seasonal and local products, enjoying socialization with meals, and encouraging other healthy habits such as exercise and water intake [5]. Adherence to this dietary pattern has been found to be associated with lower body weight, to prevent long-term weight regain, and to induce significant weight loss, with or without energy restriction [6]. Weight gain and body mass are central to the rising incidence of type 2 diabetes through several mechanisms, inducing inflammation and oxidative stress which may lead to β-cell damage and dysfunction [7]. Adherence to the Mediterranean diet also contributes directly to better metabolic control, including an improvement in insulin sensitivity and lipid profile [3].

Glycated hemoglobin (HbA1c) is frequently used as a biomarker to monitor glycemic control in type 2 diabetes. Nevertheless, HbA1c indicates the average glycemia over approximately the previous 2–3 months [8], which means that it can only reflect the efficacy of a dietary intervention after this time period.

There is evidence of an association between gut microbiota and diabetes [9]. A typical diabetogenic diet, the Western diet, rich in processed foods, salt, sugar, and saturated fat and poor in fiber is associated with an imbalance in the bacterial community, particularly a decrease in bacterial richness and diversity, which is known as dysbiosis [10,11]. Gut dysbiosis contributes to low-grade inflammation by altering gastrointestinal barrier function which can trigger impaired insulin resistance and promote type 2 diabetes [12]. However, the Mediterranean diet, which is rich in fiber, monounsaturated and polyunsaturated fatty acids, and phytochemicals, may correct this gut bacterial imbalance by promoting an increase in bacteria diversity, specific protective taxa (*Bifidobacterium*, *Roseburia*, and *Faecalibacterium*), and metabolites (short-chain fatty acids (acetate, propionate, and butyrate)), resulting in increased gut epithelial integrity and insulin sensitivity [10,13,14].

Even though the Mediterranean diet may be beneficial towards type 2 diabetes control, the molecular and physiological mechanisms by which this dietary pattern achieves metabolic improvement have not been fully elucidated. Because gut microbiota responds rapidly to dietary changes [15], we hypothesize that the effect of the Mediterranean diet on glucose homeostasis could be mediated by gut microbiota. This knowledge would help clinicians to develop novel strategies for the treatment of diabetes and to unravel different tools to evaluate adherence to dietary interventions [16,17].

In this regard, this pilot study aimed to characterize gut microbiota to monitor the effectiveness of Mediterranean diet intervention in type 2 diabetes after 4 weeks and 12 weeks.

## 2. Materials and Methods

### 2.1. Study Design and Participants

The MEDBIOME study was a 12-week single-arm pilot trial conducted at Faculdade de Ciências Médicas|NOVA Medical School, Universidade NOVA de Lisboa. The study was approved by the Ethics Committee of Administração Regional de Saúde de Lisboa e Vale do Tejo (Ref. 016/CES/ INV/2019) and Faculdade de Ciências Médicas|NOVA Medical School, Universidade NOVA de Lisboa (Ref. 55/2018/CEFCM). The study was conducted according to the Good Clinical Practice guidelines (Declaration of Helsinki) and applicable national law. This trial is registered at clinicaltrials.gov as NCT04403217.

The detailed study protocol has been published elsewhere [18]. Key inclusion criteria were men and women aged 40 to 80 years, nonsmokers, with diagnosis of type 2 diabetes (according to American Diabetes Association (ADA) criteria [19]), willing and able to provide informed consent. Participants were excluded if they had been diagnosed with diabetes before 40 years old (to exclude subjects with early-onset diabetes and maturity-onset diabetes of the young (MODY)), had changes in oral glycemic-control medications in the last 3 months, had HbA1c levels under 6.4% or above 10%, were under insulin therapy and/or corticotherapy, had triacylglyceride levels above 4.52 nmol/L (400 mg/dL), had intake of antibiotics in the last 12 weeks, or were diagnosed with any digestive disease including functional bowel disorders such as irritable bowel syndrome. Participants were referenced by physicians from different health centers of ACES Lisboa Central.

### 2.2. Intervention

Participants received an individualized structured dietary plan considering participants’ dietary history and nutritional needs. This was achieved using a food-based diet with recommendations based on the Portuguese Mediterranean Food Wheel [20]. The guide provides recommendations on the average number of standard servings of the seven core food groups (vegetables; fruits; grains and cereals; fish, meat, and eggs; legumes; dairy products; and fats) that an individual should consume to meet nutritional requirements based on age and sex. Dietary plans were elaborated using the software Nutrium. For participants with excess weight, it was decided to restrict 500–750 kcal/day of the energy requirements, never lower than 1200 kcal/day for women and 1500 kcal/day for men [21]. The distribution of macronutrients followed the recommendations of American Diabetes Association (ADA) (26–45% of total energy from carbohydrates and 20–30% of total energy from protein) [21] with 1:3 soluble to insoluble fiber ratio [22]. Adherence to the prescribed diet was monitored through 24-h dietary recalls (two nonconsecutive, 15 days apart), previously validated in the Portuguese population [23]. In addition, Mediterranean Diet Adherence Screener (MEDAS) was used to evaluate the adherence to the Mediterranean Diet [24]. MEDAS score was grouped into high adherence (score ≥ 10) or low adherence (score < 10) [25].

To increase adherence to the diet, participants attended individual dietary appointments approximately every 2 weeks. The outcome variables were measured for study participants at baseline and 4 and 12 weeks after the intervention.

### 2.3. Outcomes

#### 2.3.1. Anthropometric Measurements and Body Composition

Height was measured with a scale-mounted stadiometer and waist circumference was measured using a measuring tape according to the Directorate-General for Health [26]. The participants’ body composition was assessed while in fasting state (10–12 h) using Inbody model 770, according to the manufacturer’s specifications.

#### 2.3.2. Blood Pressure Assessment

Systolic and diastolic blood pressure was measured with a sphygmomanometer according to the protocol from the Directorate-General for Health [27].

#### 2.3.3. Biochemical Parameters

Venous blood samples were collected while the participants were in fasting state (10–12 h) by venepuncture into sterile serum separator tubes (BD Vacutainer SST II Advance; Becton, Dickinson and Company, Porto, Portugal). For HbA1c measurement, blood was collected into sterile tubes containing K_2_EDTA (EDTA BD Vacutainer; Becton, Dickinson and Company, Porto, Portugal). All blood samples were sent to an outsourced certified medical laboratory (Unilabs, S.A., Porto, Portugal) under refrigerated conditions.

Fasting glucose, triacylglycerides, total cholesterol, high-density lipoprotein (HDL) cholesterol, very low-density lipoprotein (VLDL) cholesterol, iron, urea, uric acid, creatinine, total protein, albumin, aspartate aminotransferase, alanine aminotransferase, gamma-glutamyl transferase, cholecystokinin, and serum alkaline phosphatase (ALP) were measured by specific enzymatic colorimetric methods. HbA1c was measured by quantitative high-performance liquid chromatography, and insulin was quantified by chemiluminescence immunoassay. In addition, C-reactive protein was measured by immunoturbidimetry, and sodium, potassium, and chloride were determined by selective electrode potentiometry. Low-density lipoprotein (LDL) cholesterol was estimated by the Friedewald formula [28]. Insulin resistance and sensitivity and β-cell function were estimated by Homeostatic Model Assessment (HOMA) [29].

#### 2.3.4. Gut Microbiota Composition

Fecal samples were collected into sterile tubes containing RNAlater by the participants for gut microbiota composition. The consistency of the sample was classified according to the Bristol stool scale [30]. Genomic DNA was extracted and purified from fecal samples using NZY Tissue gDNA Isolation Kit (NZY Tech, Lisboa, Portugal), as previously described by Marques et al. [31]. Libraries were processed and sequenced following the 16S Metagenomic Sequencing Library Preparation protocol from Illumina (Illumina; San Diego, CA, USA). A set of primers was used to capture the region V3–V4 of the bacterial 16S rRNA gene. The samples were pooled and loaded into the Illumina MiSeq System and sequenced using a 280-multiplex approach on a 2 × 250 bp run, according to manufacturer’s procedures [32]. The taxonomy of each sample was determined using the Kraken2 software and Bracken software, using our custom 16S database (GutHealth_DB) based on a combination of the NCBI Bacteria and Archaea: 16S ribosomal RNA project (https://www.ncbi.nlm.nih.gov/refseq/targetedloci/, accessed on 5 March 2020) and Greengenes 13_8 database (https://greengenes.secondgenome.com, accessed on 5 March 2020). Gut bacterial diversity was evaluated by Shannon index, and gut bacterial richness was measured by Chao1 index.

#### 2.3.5. Fecal Alkaline Phosphatase (ALP) Activity

ALP activity was determined in fecal samples as a marker of intestinal inflammation and permeability [33]. The protocol was adapted from Calhau et al. [34] and Malo et al. [35]. Briefly, 35 mg of each sample were homogenized with 1750 μL of extraction buffer (1 mM MgCl_2_ and 10 mM Tris-HCl, pH 8.0) and centrifuged at 10,000× *g* for 20 min. The supernatant was mixed thoroughly with dilution buffer (1 mM MgCl_2_ and 200 mM Tris-base, pH 10.4) at 1:1 ratio, and the tubes remained in a water bath for 10 min at 37 °C. *p*-Nitrophenylphosphate (*p*NPP) was added to a final concentration of 5 mM and incubated for 5 min, maintaining the tubes at the same temperature (37 °C). The reaction was stopped on ice with 5 mL of cold NaOH (0.02 M). Absorbance was read at 410 nm. The protein content of the fecal samples was determined using EZQ Protein Quantitation Kit (ThermoFisher, Lisboa, Portugal) according to the manufacturer’s specifications. Fecal ALP activity was expressed as U ALP/min/mg of protein.

### 2.4. Statistical Analysis

Statistical analysis was performed by SPSS V.25 software (IBM SPSS Statistics for Windows, IBM Corporation, Armonk, NY, USA). The Kolmogorov–Smirnov test was performed to test the normality of the distribution. One-way ANOVA followed by Bonferroni test was used for parametric variables to evaluate the effects of dietary intervention throughout time. For nonparametric variables, the Friedman test and repeated Wilcoxon tests were used. Categorical variables were analyzed by X^2^ test. Correlations between outcomes after 12 weeks of the intervention were analyzed by Pearson’s chi-square and Spearman correlation test. The differences were considered statistically significant when *p* < 0.05; because of the small sample size, effect sizes (Cohen d test) are also presented to decide whether a clinically relevant effect is found (|d| > 0.50 indicates a medium effect size and |d| > 0.80 indicates a large effect size). Data are presented as mean ± SD.

## 3. Results

### 3.1. Demographics and Baseline Characteristics

Between October 2018 and December 2019, a total of 56 individuals were recruited from different health centers, and 11 were eligible to participate in this study. Two participants were lost to follow-up after 3 weeks due to the restrictions imposed by the COVID-19 pandemic, and one participant dropped out after 6 weeks due to the inability to comply with the dietary intervention. Out of the nine participants that completed at least half of the study, three were female and six were male. The age ranged from 47 to 77 years with the mean age of 66 ± 9 years. All but one participant took oral glucose-lowering drugs, and their therapies remained unchanged during the course of this study. Additionally, eight of the participants had a diagnosis of hypertension, and five of the participants had a diagnosis of dyslipidemia (Table 1).

### 3.2. Dietary Habits

MEDAS score increased from 8.11 ± 2.26 to 10.90 ± 1.25 after 12 weeks of Mediterranean diet intervention (*p* < 0.05; Cohen d = 1.58).

**Figure 1:**
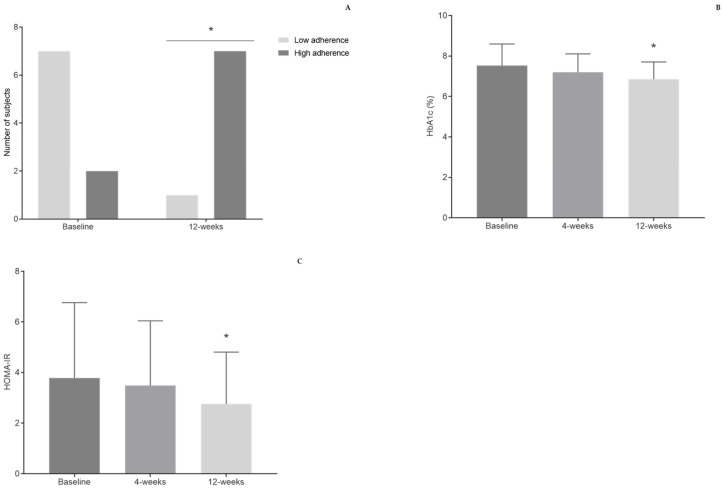
Adherence to the Mediterranean diet after 12 weeks. Low adherence—MEDAS score < 10; high adherence—MEDAS score ≥ 10 (**A**). Changes in HbA1c (**B**) and HOMA-IR (**C**) after 4 weeks and 12 weeks of the intervention. HbA1c—glycated hemoglobin; HOMA-IR—Homeostatic Model Assessment for Insulin Resistance. * Differences were considered statistically significant when *p* < 0.05 vs. baseline.

Moreover, the proportion of participants with high adherence to the Mediterranean diet increased from 22.22% to 87.50% (*p* < 0.05) at the end of the study (Figure 1A). To identify what resulted in a higher adherence to this diet, the questions from MEDAS questionnaire were analyzed individually. An increase was observed in the proportion of subjects that consumed less than 12 g of butter, margarine, or cream per day (33.33% to 100%), and an increase was observed in the proportion of subjects that consumed at least three portions (30 g) of nuts per week (33.33% to 100%) (*p* < 0.05). In addition, the proportion of participants that consumed at least 4 spoons of olive oil per day tended to increase (33.33% to 75.00%, *p* = 0.086), as did the proportion of participants that consumed less than one portion (100–150 g) of red or processed meat per day (66.67% to 100%, *p* = 0.072) (Table 2).

The 24-h dietary recall records collected were inserted in the Food Processor Program. The main results of participants’ dietary intake are presented in Supplementary Table S1. In terms of macronutrient distribution, the percentage of energy from fat changed after 4 weeks (from 9.27 ± 4.64% to 22.50 ± 5.12%, *p* < 0.05; Cohen d = 2.72) and 12 weeks (from 9.27 ± 4.64% to 19.38 ± 6.87%, *p* < 0.05; Cohen d = 1.76) of the dietary intervention. Only the percentage of energy from protein was within the recommended range. Results also showed a decrease in trans fats from 0.84 ± 0.17 g to 0.15 ± 0.28 g by the end of the study (*p* < 0.05; Cohen d = −6.45).

### 3.3. Body Composition, Anthropometric Evaluation, and Blood Pressure

There were no differences in body composition, anthropometric measures, or blood pressure after the intervention (Table 3). However, diastolic blood pressure was lower in subjects with high adherence to the Mediterranean diet (low adherence—85.63 ± 8.14 mmHg vs. high adherence—75.48 ± 7.45 mmHg, *p* < 0.05).

### 3.4. Biochemical Parameters

Data on changes in glucose homeostasis and lipid profile are shown in Table 3 (see Supplementary Table S2 for other biochemical parameters).

Time since diagnosis of diabetes was not correlated with glycemic profile at baseline and did not interfere with the impact of the intervention (*p* > 0.05).

Twelve weeks after the intervention, HbA1c decreased by 0.67% (7.53 ± 1.07% to 6.86 ± 0.85%, *p* < 0.05; Cohen d = −0.70) and HOMA-IR decreased from 3.79 ± 2.98 to 2.76 ± 2.05 (*p* < 0.05; Cohen d = −0.41), with no accompanying changes in lipid profile (Figure 1B, C).

### 3.5. Gut Microbiota

**Figure 2:**
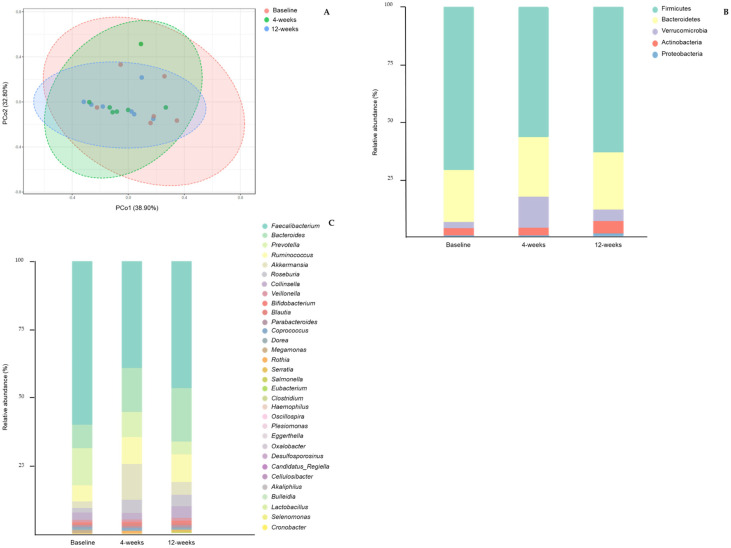
Clustering of fecal bacterial genera communities by principal component analysis (PCoA) (**A**). Data are plotted according to the first two principal components, which explain 32.80% (PCo1) and 38.90% (PCo2) of gut microbiota composition variation at 4 weeks and 12 weeks of the intervention. Each point represents one sample. Gut microbiota composition considering phylum (**B**) and genera (**C**) level at baseline, 4 weeks, and 12 weeks after intervention. Bars represent the average relative abundance of each bacterial phylum/genus. Each phylum/genus is represented by a different color.

A principal component analysis plot of Bray–Curtis index distance was applied for the visualization of the complex relationships of gut microbiota composition at each time point (baseline, 4 weeks, and 12 weeks). No differences were observed, suggesting similarity among the groups clustered together (Figure 2A, *p* = 0.639).

Participants’ gut microbiota composition (phylum and genus level) according to collection time is presented in Figure 2B,C. Clustering of bacteria genera and species according to Mediterranean diet adherence is shown in Supplementary Figure S1.

**Figure 3:**
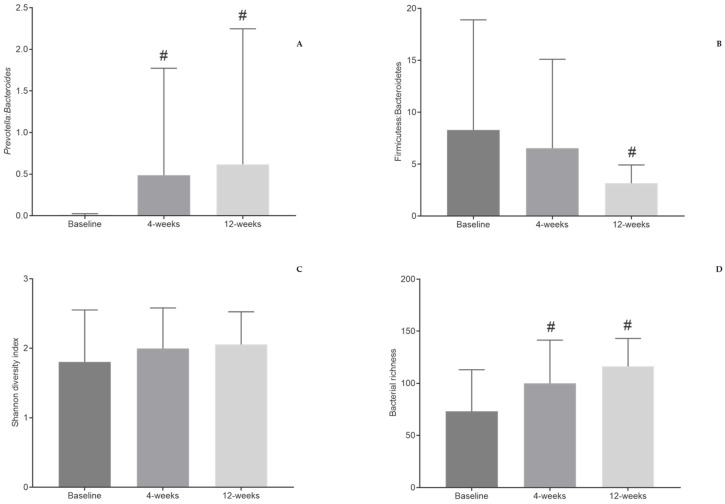
Changes in *Prevotella* to *Bacteroides* ratio (**A**), Firmicutes to Bacteroidetes ratio (**B**), bacterial diversity (**C**), and bacterial richness (**D**) after 4 weeks and 12 weeks of the intervention. # Differences were considered clinically relevant when |d| > 0.50 vs. baseline.

*Prevotella* to *Bacteroides* ratio tended to increase right after 4 weeks of the intervention (0.01 ± 0.02 to 0.49 ± 1.29 at 4 weeks and 0.62 ± 1.63 at 12 weeks, *p* = 0.438; 4 weeks Cohen d = 0.74 and 12 weeks Cohen d = 0.74) (Figure 3A).

The effect size induced by the Mediterranean diet on the Firmicutes to Bacteroidetes ratio was considered clinically relevant after 12 weeks (8.30 ± 10.61 to 3.18 ± 1.75, *p* = 0.846; Cohen d = −0.83) (Figure 3B).

No differences were observed in bacterial diversity by the end of the intervention (1.80 ± 0.75 to 2.05 ± 0.47, *p =* 0.449; Cohen d = 0.40) (Figure 3C). Gut bacterial richness tended to increase from baseline to 4 and 12 weeks after the intervention (73.14 ± 39.91 to 100.00 ± 41.47 at 4 weeks and 116.30 ± 26.73 at 12 weeks, *p* = 0.205; 4 weeks Cohen d = 0.66 and 12 weeks Cohen d = 1.29) (Figure 3D).

**Figure 4:**
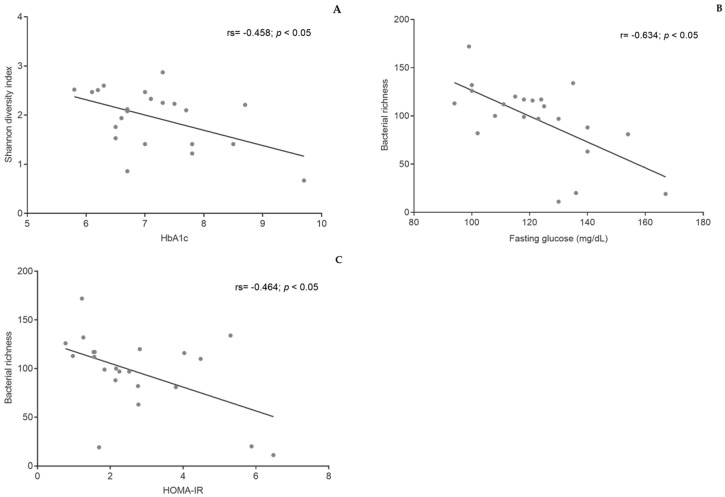
Correlations between bacterial diversity and HbA1c (**A**), bacterial richness and fasting glucose (**B**), and bacterial richness and HOMA-IR (**C**). HbA1c—glycated hemoglobin; HOMA-IR—homeostatic model assessment of insulin resistance.

Additionally, bacterial diversity was negatively correlated with HbA1c (rs = −0.458 *p* < 0.05), and bacterial richness was negatively correlated with fasting glucose levels (r = −0.634; *p* < 0.05) and HOMA-IR (rs = −0.464; *p* < 0.05) (Figure 4).

### 3.6. ALP Activity in Fecal Samples

**Figure 5:**
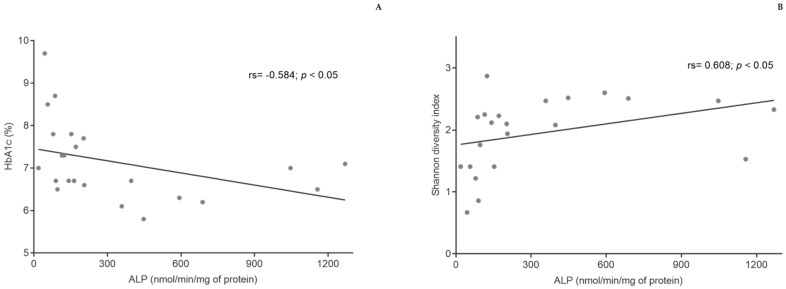
Correlations between HbA1c and ALP (**A**) and bacterial diversity and ALP (**B**). HbA1c—glycated hemoglobin; ALP—alkaline phosphatase.

Although no changes were observed in ALP activity from fecal samples after the intervention (data not shown), the activity of this enzyme was negatively correlated with HbA1c (rs = −0.584; *p* < 0.05) and positively correlated with bacterial diversity (rs = 0.608; *p* < 0.05) (Figure 5).

## 4. Discussion

The MEDBIOME study reinforces the role of the Mediterranean diet in metabolic control of subjects with type 2 diabetes and highlights that this effect goes beyond mere body weight loss or total energy intake. Additionally, the medication was not altered during the 3 months prior to the beginning of the study and remained the same during the intervention, suggesting that the metabolic impact observed was mainly due to the Mediterranean diet adherence.

According to a systematic review with meta-analysis of four interventional trials [36], the Mediterranean diet results in a reduction ranging from 0.30% to 0.47% of HbA1c compared with control diet. Nonetheless, the results of our study showed a decrease of 0.67%. In addition, HbA1c levels were below 7.00% after the intervention, a glycemic target value in subjects with diabetes that is associated with a decrease in microvascular complications [8]. The magnitude of effect in this study may be attributable to the fortnightly follow-up where the participants were given dietary advice promoting Mediterranean diet yet individually tailored to their dietary preferences, which facilitated the participants’ adherence to this diet and resulted in a higher metabolic impact. It is important to note that the promotion of dietary interventions will have an impact not only on individual health outcomes but also on direct and indirect costs related to type 2 diabetes, which will alleviate the global economic burden of this condition that is projected to increase from $1.3 trillion worldwide to almost double that in 2030 [37].

Adherence to the Mediterranean diet has increased mainly due to the replacement of the type of fat consumed by the participants, namely the replacement of foods richer in trans and saturated fatty acids with foods richer in monounsaturated and polyunsaturated fatty acids. Monounsaturated fatty acids are thought to counteract the effect of saturated fatty acids, which increases insulin resistance of peripheral tissues, and polyunsaturated fatty acids are known to reduce inflammation, also resulting in an improvement in peripheral insulin responsiveness [38]. This is in line with the results of this study because HOMA-IR was reduced after the intervention.

Furthermore, these results are in accordance with recent studies showing that subjects with type 2 diabetes have an unbalanced gut microbiota [12]. Regarding the enterotype, *Prevotella* to *Bacteroides* ratio tended to increase starting from 4 weeks. This ratio is usually lower in subjects with type 2 diabetes according to Larsen et al. [39] and Wang et al. [40], which results in higher metabolic endotoxemia and inflammation [39]. *Prevotella*-dominated microbiota is associated with an increased capacity to ferment complex polysaccharides [40], producing 2–3 times more propionate than *Bacteroides*-dominated microbiota [41], and has been previously linked to the Mediterranean diet [42,43].

In comparison to nondiabetic Portuguese individuals that have a bacterial diversity ranging from 2.70 to 3.00 and a Firmicutes to Bacteroidetes ratio ranging from 0.89 to 1.32 (data not published) [44], our results show that at baseline the individuals with type 2 diabetes have lower bacterial diversity and a higher Firmicutes to Bacteroidetes ratio. Nevertheless, the results of this study indicate that the Mediterranean diet may result in several beneficial modifications towards gut microbiota composition right after 4 weeks. These results are corroborated by findings from Filippis et al. [45], who observed a beneficial gut-related metabolome profile associated with higher adherence to the Mediterranean diet. In our case, an increase was observed in both bacterial diversity and richness, and a decrease was observed in Firmicutes to Bacteroidetes ratio; together, these factors promote gut homeostasis [11].

The balance in gut microbiota is associated with the production of short-chain fatty acids that can bind to the G protein-coupled receptors GPCR-41 and GPCR-43 and induce secretion of glucagon-like peptide 1, contributing to increased energy expenditure, decreased food intake, improved glucose metabolism and insulin secretion, and better intestinal barrier function [43,46,47]. Consequently, improved intestinal barrier function may reduce the translocation of bacteria and lipopolysaccharides, which increases anti-inflammatory markers and decreases proinflammatory markers and HbA1c [48]. Therefore, the Mediterranean diet has a more direct impact on gut microbiota, preceding the metabolic effect similar to what has been described after bariatric surgery [49].

To analyze intestinal permeability and inflammation, ALP activity from fecal samples was determined. ALPs are a group of metalloenzymes that catalyze the removal of phosphate groups [50]. There are four isoenzymes that have different metabolic functions; one of these isoenzymes is intestinal ALP, an isoenzyme that according to Malo et al. [35] represents most of the ALP activity from fecal samples. Intestinal ALP is strongly related to diet quality; it has been shown that an increase in consumption of fiber, polyphenols, and polyunsaturated fats, which is praised in the Mediterranean diet, may enhance the activity of intestinal ALP [51,52,53]. Although no differences were observed in ALP activity from fecal samples after Mediterranean diet intervention, the activity of this enzyme was correlated with an increase in bacteria diversity and a decrease in HbA1c. In fact, it was previously observed that the increase in intestinal ALP reduces the risk of type 2 diabetes and that the activity of the enzyme is actually lower in subjects with this disease [35]. This may be explained by the ability of intestinal ALP to attenuate inflammation by modifying gut microbiota composition and by dephosphorylating lipopolysaccharides, reducing gut permeability, and increasing insulin sensitivity [50,53].

Although the results of this study are encouraging, there are some limitations that should be considered. A major limitation of the study was the lack of a control group. That said, because the importance of diet in the treatment of type 2 diabetes is well established, it would be unethical to have a group without any dietary intervention. Moreover, the participants were all referred from health centers without registered dietitians that could provide dietary intervention and also had lower adherence to the Mediterranean diet before the intervention. Hence, these patients can be considered their own “controls” because they were not achieving good metabolic control with the standard pharmacologic therapy and without any dietary recommendations to follow a specific dietary pattern, as shown by the HbA1c levels at baseline. Thus, despite the lack of a control group, it is unlikely that the effects observed in the glycemic control could be derived only from other factors not related to Mediterranean diet intervention. Another important limitation is that the dietary intake was self-reported, a method described to be imprecise, although the dietitian was trained to reduce this bias by using strategies such as the food quantification manual, food labels, and asking participants to take photos of the food whenever necessary. It is also important to consider the need to standardize operational procedures for the analysis of the microbiota, namely sequencing methodology and bioinformatics analysis, to allow proper comparisons. Lastly, the study’s small sample size was a major limitation and resulted in lower statistical power. This was mostly caused by the difficulty in recruitment due to high nonacceptance rates during the recruitment phase and the established eligibility criteria, which were strictly narrow in order to guarantee a homogeneous group at baseline and avoid potential confounders that could mask the effects of the dietary intervention. Nonetheless, even with a small number of participants, it was possible to observe statistically and clinically relevant differences in the main outcomes of the study after the intervention.

## 5. Conclusions

In conclusion, the MEDBIOME study strengthens the role of dietary intervention in type 2 diabetes management, indicating the need to employ more dietitians to provide efficient nutritional and dietary support in primary healthcare centers and thus contribute to increasing the quality of life of these patients. This study also suggests that the Mediterranean diet is effective in improving metabolic control of subjects with type 2 diabetes, independently of energy intake and weight loss. Additionally, gut microbiota changes appear to precede the changes in standard biomarkers of type 2 diabetes (HbA1c), suggesting that the effects of MD in type 2 diabetes are mediated by the gut microbiota. In addition, the results of this study highlight the importance of gut microbiota composition, in particular gut bacterial richness, which could be used as a new biomarker for early measurement of the effectiveness of dietary interventions in the metabolic control of type 2 diabetes.

## Figures and Tables

**Table 1:** Baseline characteristics.

	Baseline Characteristics ^1^
	(*n* = 9)
Sex	
Male	6 (66.67%)
Female	3 (33.33%)
Age (years)	66 (47–77)
Race	
Caucasian	9 (100%)
Other complications	
Hypertension	8 (88.89%)
Dyslipidemia	5 (55.56%)
Cardiovascular disease	1 (11.11%)
Time since diabetes diagnosis (years)	13.63 (5–23)
Oral antidiabetic drugs	
Biguanides	8 (88.89%)
Dipeptidyl peptidase-4 inhibitors	3 (33.33%)
SGLT2-inhibitors	1 (11.11%)
Sulfonylurea	1 (11.11%)

^1^ Data are expressed as *n* (%). Age and time since diabetes diagnosis are expressed as mean (minimum–maximum).

**Table 2:** Food group consumption evaluated by MEDAS questionnaire.

	Baseline ^1^	12 Weeks ^1^	*p* Value
	(*n* = 9)	(*n* = 8)	
Vegetables (≥2 portions/day)	4 (44.44%)	6 (75.00%)	0.201
Fruit (≥3 portions/day)	4 (44.44%)	6 (75.00%)	0.201
Legumes (≥3 portions/week)	5 (55.56%)	3 (37.50%)	0.457
Fish and seafood (≥3 portions/week)	7 (77.78%)	6 (75.00%)	0.893
Red meat (<1 portion/day)	6 (66.67%)	8 (100%)	0.072
Butter, margarine, and cream (<1 portion/day)	3 (33.33%)	8 (100%)	0.004 *
Pastries (<3 portions/week)	7 (77.78%)	8 (100%)	0.156
Nuts (≥3 portions/week)	3 (33.33%)	8 (100%)	0.004 *
Sugary drinks—soft drinks and juices (<1 drink/day)	8 (88.89%)	8 (100%)	0.331
Wine (≥7 cups/week)	5 (55.56%)	2 (25.00%)	0.201
Olive oil (≥4 spoons/day)	3 (33.33%)	6 (75.00%)	0.086

^1^ Data are expressed as *n* (%). * *p* < 0.05 vs. baseline.

**Table 3:** Anthropometric, body composition, and blood pressure measurements and evaluation of biochemical parameters (glucose homeostasis and lipid profile).

	Baseline ^1^	4 Weeks ^1^	12 Weeks ^1^	*p* Value
	(*n* = 8–9)	(*n* = 8–9)	(*n* = 8)	
Weight (kg)	76.39 ± 17.79	77.52 ± 16.94	73.69 ± 14.48	0.206
Waist perimeter (cm)	94.44 ± 12.25	95.17 ± 12.67	93.36 ± 11.41	0.501
Waist-to-hip ratio	0.93 ± 0.05	0.93 ± 0.07	0.93 ± 0.06	0.867
Body mass index (kg/m^2^)	27.60 ± 4.03	27.09 ± 3.75	26.95 ± 3.84	0.056
Body fat mass (kg)	22.41 ± 6.91	23.14 ± 7.45	23.56 ± 6.80	0.093
Free fat mass (kg)	52.49 ± 10.69	53.01 ± 10.38	50.13 ± 8.84	0.908
Skeletal muscle mass (kg)	27.69 ± 5.57	28.05 ± 5.46	27.59 ± 5.35	0.578
Systolic blood pressure (mmHg)	141.71 ± 12.40	142.33 ± 13.04	136.54 ± 12.08	0.227
Diastolic blood pressure (mmHg)	82.25 ± 9.98	83.54 ± 11.38	79.98.0 ± 8.02	0.496
Glucose homeostasis and lipid profile parameters				
Fasting glucose (mg/dL)	131.63 ± 8.53	127.38 ± 7.69	122.50 ± 9.42	0.581
Insulin (μU/mL)	11.26 ± 2.65	10.80 ± 2.31	8.48 ± 1.60	0.157
HbA1c (%)	7.53 ± 1.07	7.20 ± 0.91	6.86 ± 0.85 *	0.024 *
HOMA-IR	3.79 ± 2.98	3.49 ± 2.56	2.76 ± 2.05 *	0.044 *
HOMA-B	61.23 ± 10.41	57.68 ± 9.39	51.82 ± 6.06	0.512
HOMA-S (%)	39.34 ± 7.87	48.44 ± 14.44	52.71 ± 10.52	0.134
Total cholesterol (mg/dL)	172.44 ± 32.77	162.11 ± 15.33	168.25 ± 20.46	0.479
LDL cholesterol (mg/dL)	93.56 ± 24.59	87.44 ± 17.04	90.50 ± 19.70	0.688
VLDL cholesterol (mg/dL)	29.00 ± 16.23	27.89 ± 17.72	28.00 ± 15.18	0.135
HDL cholesterol (mg/dL)	49.89 ± 18.20	47.11 ± 17.42	50.00 ± 15.50	0.459
Triacylglycerides (mg/dL)	145.33 ± 81.58	139.00 ± 89.34	140.38 ± 76.71	0.163

^1^ Data are expressed as mean ± SD. HbA1c—glycated hemoglobin; HOMA-IR—Homeostatic Model Assessment for Insulin Resistance; HOMA-B—Homeostatic Model Assessment for β-cell function; HOMA-S—Homeostatic Model Assessment for Insulin Sensitivity; LDL—low-density lipoprotein; VLDL—very low-density lipoprotein; HDL—high-density lipoprotein. * *p* < 0.05 vs. baseline.

## Data Availability

All relevant data are within the paper and its Supplementary Materials.

## Supplementary Materials

The following are available online at https://www.mdpi.com/article/10.3390/nu13041228/s1: Table S1: Macronutrient energy percentage and macronutrients and micronutrients ingested quantity. Table S2: Biochemical parameters. Figure S1: Clustering of bacterial genera (A) and species (B) according to Mediterranean diet adherence visualized as a heatmap.
