# Supplementary material for: A Pilot Study on the Metabolic Impact of Mediterranean Diet in Type 2 Diabetes: Is Gut Microbiota the Key?
Source: Nutrients. 2021 Apr 8;13(4):1228. doi: 10.3390/nu13041228 (PMC8068165; doi:10.3390/nu13041228)
Supplement: Supplementary file 1 [file nutrients-13-01228-s001.zip › Supplementary figures.pptx]

## Slide 1
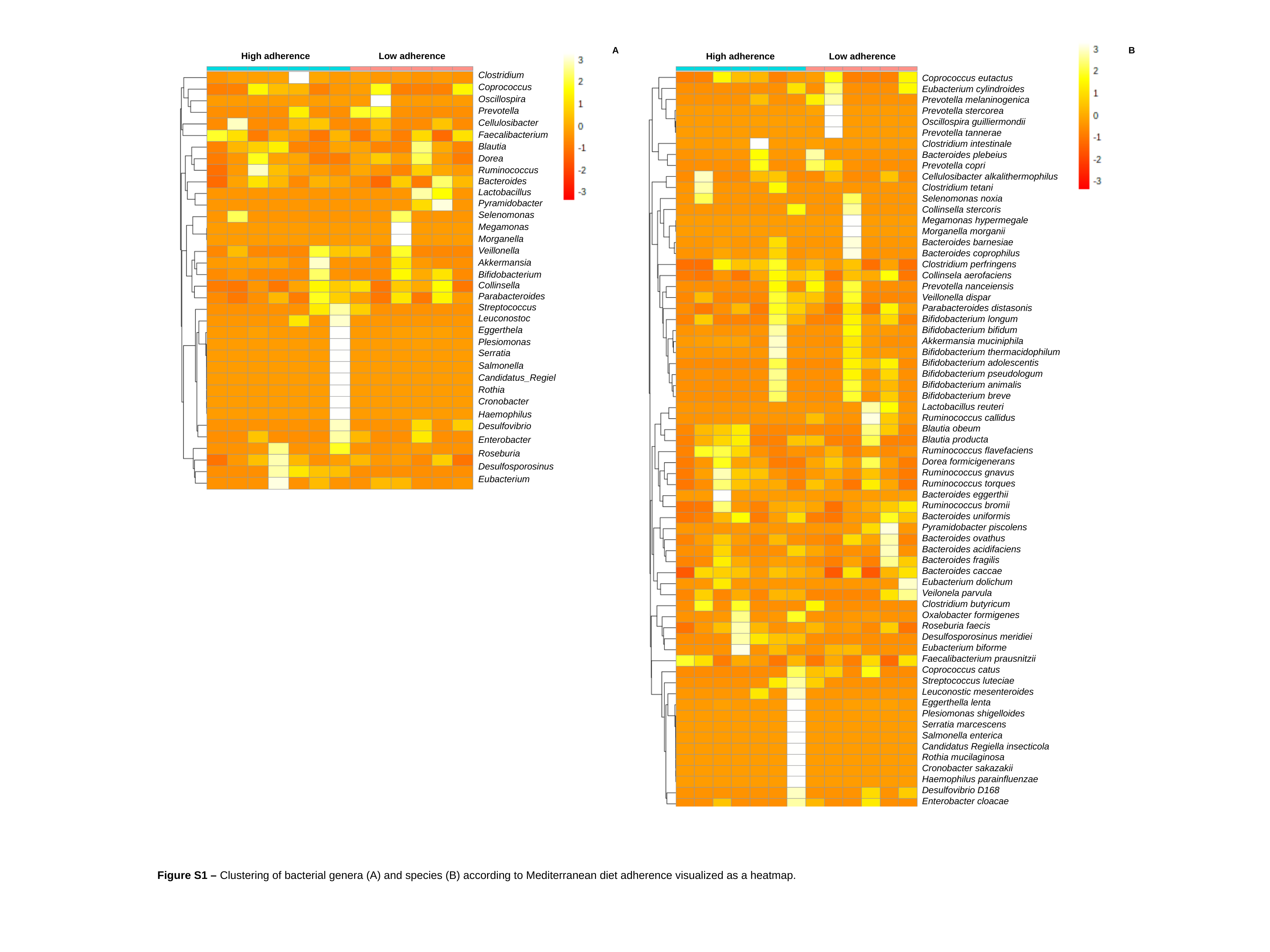

Low adherence
High adherence
Coprococcus eutactus
Eubacterium cylindroides
Prevotella melaninogenica
Prevotella stercorea
Oscillospira guilliermondii
Prevotella tannerae
Clostridium intestinale
Bacteroides plebeius
Prevotella copri
Cellulosibacter alkalithermophilus
Clostridium tetani
Selenomonas noxia
Collinsella stercoris
Megamonas hypermegale
Morganella morganii
Bacteroides barnesiae
Bacteroides coprophilus
Clostridium perfringens
Collinsela aerofaciens
Prevotella nanceiensis
Veillonella dispar
Parabacteroides distasonis
Bifidobacterium longum
Bifidobacterium bifidum
Akkermansia muciniphila
Bifidobacterium thermacidophilum
Bifidobacterium adolescentis
Bifidobacterium pseudologum
Bifidobacterium animalis
Bifidobacterium breve
Lactobacillus reuteri
Ruminococcus callidus
Blautia obeum
Blautia producta
Ruminococcus flavefaciens
Dorea formicigenerans
Ruminococcus gnavus
Ruminococcus torques
Bacteroides eggerthii
Ruminococcus bromii
Bacteroides uniformis
Pyramidobacter piscolens
Bacteroides ovathus
Bacteroides acidifaciens
Bacteroides fragilis
Bacteroides caccae
Eubacterium dolichum
Veilonela parvula
Clostridium butyricum
Oxalobacter formigenes
Roseburia faecis
Desulfosporosinus meridiei
Eubacterium biforme
Faecalibacterium prausnitzii
Coprococcus catus
Streptococcus luteciae
Leuconostic mesenteroides
Eggerthella lenta
Plesiomonas shigelloides
Serratia marcescens
Salmonella enterica
Candidatus Regiella insecticola
Rothia mucilaginosa
Cronobacter sakazakii
Haemophilus parainfluenzae
Desulfovibrio D168
Enterobacter cloacae
Low adherence
High adherence
Clostridium
Coprococcus
Oscillospira
Prevotella
Cellulosibacter
Faecalibacterium
Blautia
Dorea
Ruminococcus
Bacteroides
Lactobacillus
Pyramidobacter
Selenomonas
Megamonas
Morganella
Veillonella
Akkermansia
Bifidobacterium
Collinsella
Parabacteroides
Streptococcus
Leuconostoc
Eggerthela
Plesiomonas
Serratia
Salmonella
Candidatus_Regiel
Rothia
Cronobacter
Haemophilus
Desulfovibrio
Enterobacter
Roseburia
Desulfosporosinus
Eubacterium
B
A
Figure S1 – Clustering of bacterial genera (A) and species (B) according to Mediterranean diet adherence visualized as a heatmap.
